# AhR signaling activation disrupts migration and dendritic growth of olfactory interneurons in the developing mouse

**DOI:** 10.1038/srep26386

**Published:** 2016-05-20

**Authors:** Eiki Kimura, Yunjie Ding, Chiharu Tohyama

**Affiliations:** 1 Laboratory of Environmental Health Sciences, Center for Disease Biology and Integrative Medicine, Graduate School of Medicine, The University of Tokyo, Tokyo 113-0033, Japan; 2 Experimental Biology Laboratory, Faculty of Medicine, University of Tsukuba, Tsukuba 305-8575, Japan

## Abstract

Perinatal exposure to a low level of dioxin, a ubiquitous environmental pollutant, has been shown to induce abnormalities in learning and memory, emotion, and sociality in laboratory animals later in adulthood. However, how aryl hydrocarbon receptor (AhR) signaling activation disrupts the higher brain function remains unclear. Therefore, we studied the possible effects of excessive activation of AhR signaling on neurodevelopmental processes, such as cellular migration and neurite growth, in mice. To this end, we transfected a constitutively active-AhR plasmid into stem cells in the lateral ventricle by *in vivo* electroporation on postnatal day 1. Transfection was found to induce tangential migration delay and morphological abnormalities in neuronal precursors in the rostral migratory stream at 6 days post-electroporation (dpe) as well as disrupt radial migration in the olfactory bulb and apical and basal dendritic growth of the olfactory interneurons in the granule cell layer at 13 and 20 dpe. These results suggest that the retarded development of interneurons by the excessive AhR signaling may at least in part explain the dioxin-induced abnormal behavioral alterations previously reported in laboratory animals.

The aryl hydrocarbon receptor (AhR), a ligand-activated transcription factor that belongs to the basic helix-loop-helix (bHLH) superfamily, is present in cells of various organs, and it acts as a receptor that responds to ligands to induce downstream signaling via the expression of AhR-target genes, such as *Cyp1a1*^1^. Homologs of this gene are ubiquitously present in a variety of animal species, ranging from *Caenorhabditis elegans* to mammals, and it is evolutionally conserved^2^,^3^,^4^; hence, this gene is considered to play an essential role(s) in physiological processes. For example, a null mutation of this gene was found to impair neuronal growth and development in *C. elegans*^2^,^5^, control the dendritic arborization of sensory neurons in *Drosophila*^3^, and display a patent ductus venosus and smaller livers in mice^6^ as well as reproductive defects in male and female mice^7^,^8^. In addition, adult AhR-null mice exhibit reduced cell division, decreased cell survival, and diminished neuronal differentiation in the dentate gyrus of the hippocampus^4^. Many studies of natural ligands for AhR have identified candidate molecules, such as tryptophan derivatives^9^,^10^,^11^, but the physiological role of AhR signaling during brain development is poorly understood.

Dioxin, a ubiquitous environmental contaminant, is known to be an extremely potent exogenous AhR ligand, which causes a variety of toxic effects, such as cancer, reproductive toxicity, and immunotoxicity^12^. The manifestations of these toxicities occur in an AhR-dependent manner, as shown by experiments using AhR-deficient mice^13^,^14^,^15^. Adult rodent offspring born to dams exposed to dioxin exhibit cognitive and behavioral abnormalities, such as behavioral flexibility, paired associate learning and memory, anxiety, and sociality^16^,^17^,^18^,^19^,^20^,^21^,^22^, as well as abnormalities in dendritic growth and spine density in the pyramidal neurons^23^. *In situ* experiments to localize AhR and its dimerizing partner ARNT showed the expression of mRNA in various regions of the rat brain^24^. Thus, it is reasonable to speculate that abnormal AhR signaling links behavioral abnormalities with retarded morphology as the underlying mechanism of developmental neurotoxicity of dioxin. Because AhR-null mice have malformations, such as prolonged extramedullary hematopoiesis^15^, the suitability of AhR-null mice for use in experiments to investigate higher brain functions is considered to be limited. Instead, gain-of-function of AhR has been studied using transgenic mouse strains that express constitutively active (CA)-AhR, which is thought to reflect the situation of AhR ligand exposure. Indeed, the CA-AhR model animals were found to manifest signs of dioxin toxicities, such as thymic involution and liver enlargement, as well as tumors in the glandular part of the stomach^25^,^26^,^27^. AhR signaling activation by CA-AhR is functionally connected to a signaling cascade that leads to dramatic alterations in cell plasticity and increased cell motility^28^. Thus, it is reasonable to consider that AhR may be involved with physiological and toxicological roles.

During the growth and development of interneurons in the olfactory bulb (OB), the neuronal progenitors originate from stem cells located in the subventricular zone of the lateral ventricles (LVs), and they migrate tangentially along the entire extent of the rostral migratory stream (RMS) to their destination in the OB or accessory olfactory bulb (AOB). In the OB, the neuronal progenitors turn to move radially out of the RMS into the outer layers, such as the granule cell layer (GCL), where they differentiate into GABAergic interneurons and extend dendrites^29^,^30^,^31^,^32^,^33^. Thus, olfactory interneurons are considered to be a useful model for investigating brain development in terms of cellular migration and dendritic growth. A previous study showed that *C. elegans* AhR regulates the differentiation of GABAergic neurons^2^, so we employed neonatal *in vivo* electroporation of olfactory interneurons as a model of mammalian GABAergic neurons^34^, and we studied the physiological significance of AhR signaling activation for cellular migration and dendritic growth in the developing mouse brain.

## Results

### Effects of AhR signaling activation on tangential migration during development

**Figure 1:**
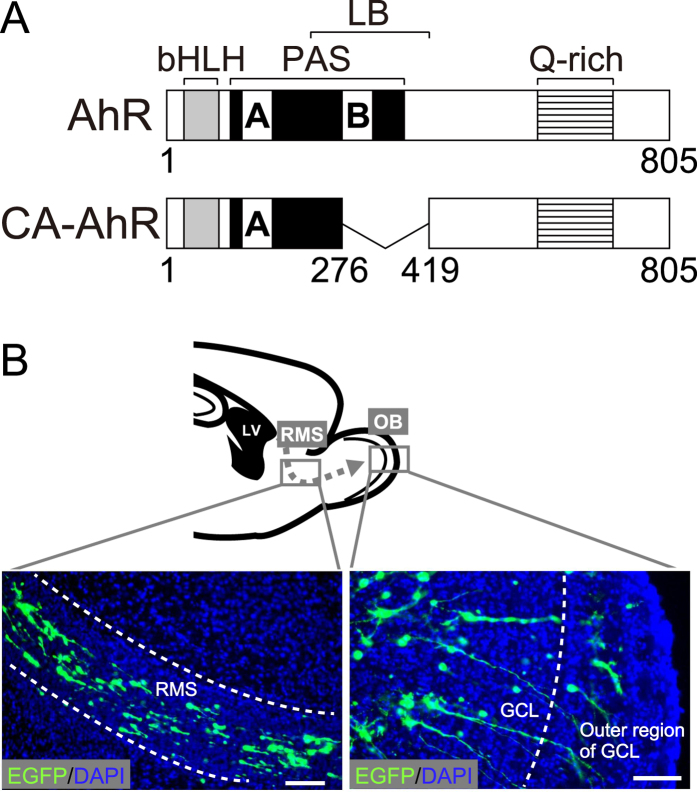
Neuronal precursor- and granule neuron-specific electroporation of pCAGGS1-EGFP and pCAGGS1-CA-AhR in the developing mouse brain. (**A**) Scheme of AhR and CA-AhR protein structures. Numbers represent the position of a given amino acid residue. (**B**) Representative photographs showing EGFP-expressing cells in the rostral migratory stream (dashed arrow) and olfactory bulb. AhR, aryl hydrocarbon receptor; CA, constitutively active; bHLH, basic helix-loop-helix domain; GCL, granule cell layer; LB, ligand-binding domain; LV, lateral ventricle; PAS, Per-Arnt-Sim domain; Q-rich, glutamine-rich domain. Scale bar = 100 μm.

**Figure 2:**
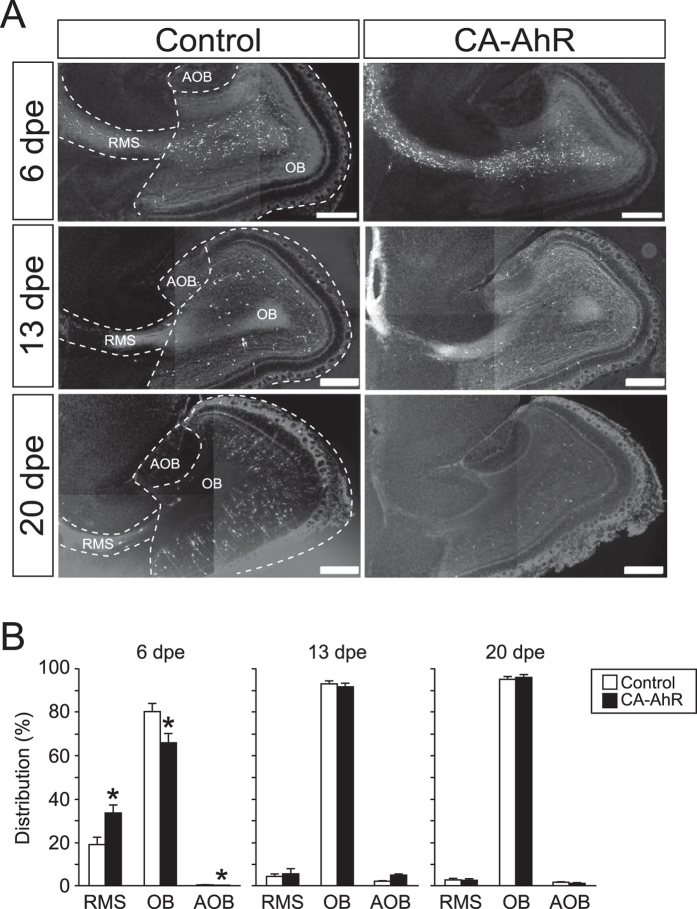
*In vivo* electroporation of CA-AhR induced the abnormal distribution of EGFP-expressing cells in the RMS, OB, and AOB of developing mice at 6, 13, and 20 dpe. (**A**) Representative photomicrographs showing EGFP-expressing cells in the RMS, OB, and AOB regions. Scale bar = 500 μm. (**B**) The distribution of EGFP-expressing cells changed significantly in the RMS, OB, and AOB in the CA-AhR group, as compared with that in the control group at 6 dpe, but not at 13 and 20 dpe. Asterisks (*): significantly different from the control group at *p* < 0.05. Values are shown as mean ± SEM for six mice/group.

To investigate the effects of AhR signaling activation on neuron development, we employed an AhR deletion mutant that lacks part of the ligand-binding domain to function as a CA-AhR (Fig. 1A)^27^, and we transfected a CA-AhR plasmid into stem cells in the LV by *in vivo* electroporation on postnatal day (PND) 1. In the brain sections from the electroporated mice, migrating EGFP-expressing cells were observed in the RMS, OB, and AOB (Fig. 1B). Next, we examined the tangential migration of neuronal precursors to the OB or AOB through the RMS of the developing brain. At 6, 13, and 20 days post-electroporation (dpe), EGFP-expressing cells were found to be present in the RMS, OB, and AOB in the control and CA-AhR groups (Fig. 2A). For the distribution of EGFP-expressing cells in the RMS, OB, and AOB, two-way repeated-measures ANOVA indicated a significant difference in the interactions of the CA-AhR effect with the distribution between the three regions (*p* < 0.001) at 6 dpe (Fig. 2B). The percentage of EGFP-expressing cells in the RMS of the CA-AhR group was significantly higher than that in the control group (CA-AhR group = 33.7% ± 3.6% vs. control group = 18.9% ± 3.6%, *p* < 0.05). The percentage in the CA-AhR group was significantly lower than that in the control group in the OB (CA-AhR group = 66.2% ± 3.6% vs. control group = 80.4% ± 3.8%, *p* < 0.05) and AOB (CA-AhR group = 0.1% ± 0.1% vs. control group = 0.7% ± 0.3%, *p* < 0.05). No significant changes in the distribution of EGFP-expressing cells were observed at 13 and 20 dpe (Fig. 2B). These results suggest that the excessive activation of AhR signaling induces a delay in the tangential migration of neuronal precursors during development.

### Effects of AhR signaling activation on the morphology of migrating precursors in the RMS at 6 dpe

**Figure 3:**
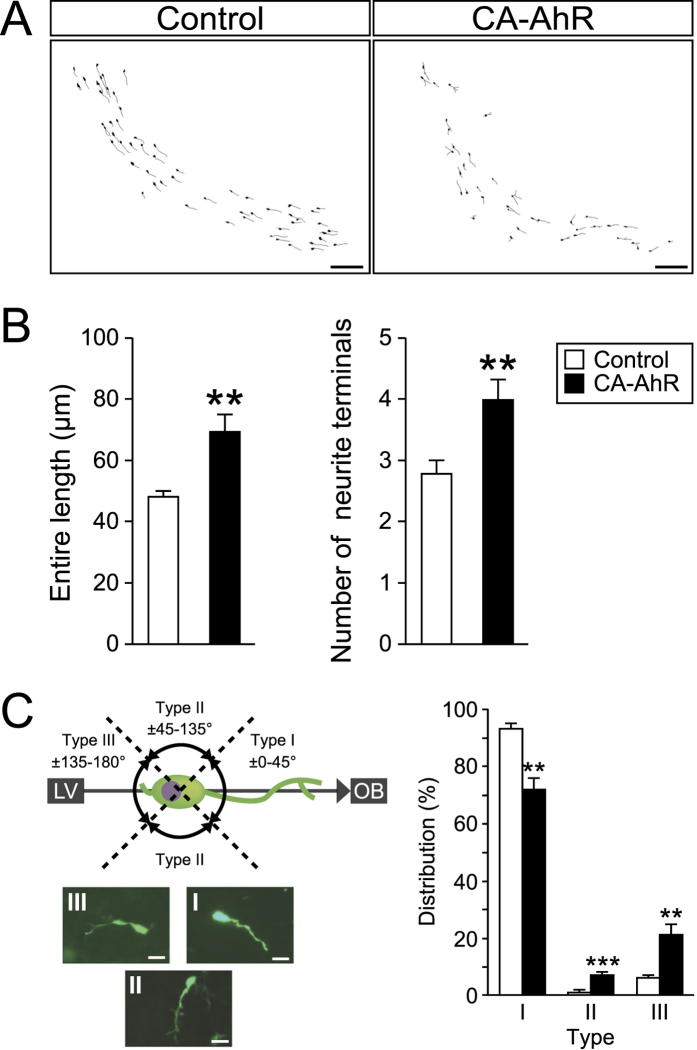
*In vivo* electroporation of CA-AhR induced abnormal morphology for EGFP-expressing cells in the RMS of developing mice at 6 dpe. (**A**) Representative images of migrating EGFP-expressing cells present in the RMS from the control and CA-AhR groups at 6 dpe. Scale bar = 100 μm. (**B**) The entire length and number of neurite terminals in EGFP-expressing cells in the RMS. (**C**) Distribution of the leading process elongation. Categorization of EGFP-expressing cells in the RMS based on the angle of orientation of the leading process relative to the RMS. Representative photographs of Type I, II, and III cells. Scale bar = 20 μm. Asterisks (*, **, and ***): significantly different from the control group at *p* < 0.05, *p* < 0.01, and *p* < 0.001, respectively. Values represent the mean ± standard error of the mean for six mice/group. AhR, aryl hydrocarbon receptor; CA, constitutively active; RMS, rostral migratory stream.

To further investigate the effects of CA-AhR on the migration of neuronal precursors, we morphometrically analyzed EGFP-expressing cells present in the RMS at 6 dpe (Fig. 3A). In the developing brain, migrating neuronal precursors possess one long neurite called the leading process and one shorter neurite called the tailing process^35^. At 6 dpe, the neuronal precursors labeled with EGFP migrated toward the OB by traversing the RMS. We analyzed the two types of neurites in EGFP-expressing cells according to the following parameters: (1) the entire length of each precursor, (2) the number of neurite terminals of each precursor, and (3) the elongation direction of the leading process. CA-AhR significantly increased the entire length and the terminal number of neurites compared with those in the control (*p* < 0.01 and <0.01, respectively; Fig. 3B). To analyze the direction of elongation for the leading process, we categorized EGFP-expressing cells in the RMS based on the angle of orientation from the leading process to the RMS. The angles of ±(0°–45°), ±(45°–135°), and ±(135°–180°) were denoted as Types I, II, and III, respectively (Fig. 3C). Two-way repeated-measures ANOVA detected a significant difference in the interactions between the CA-AhR effect and the distributions of Types I, II, and III (*p* < 0.0001). The percentage belonging to Type I was significantly lower in the CA-AhR group compared with that in the control group (CA-AhR group = 72.2% ± 4.3% vs. control group = 93.3% ± 1.8%, *p* < 0.01). By contrast, the percentages in the CA-AhR group were significantly higher compared with that in the control group for Type II (CA-AhR group = 7.1% ± 1.1% vs. control group = 0.7% ± 0.7%, *p* < 0.001) and Type III (CA-AhR group = 20.7% ± 4.3% vs. control group = 6.0% ± 1.5%, *p* < 0.01; Fig. 3C). These results suggest that the excessive activation of AhR signaling disrupts the leading process growth as well as precursor migration.

### Effects of AhR signaling activation on radial migration in the OB at 13 and 20 dpe

**Figure 4:**
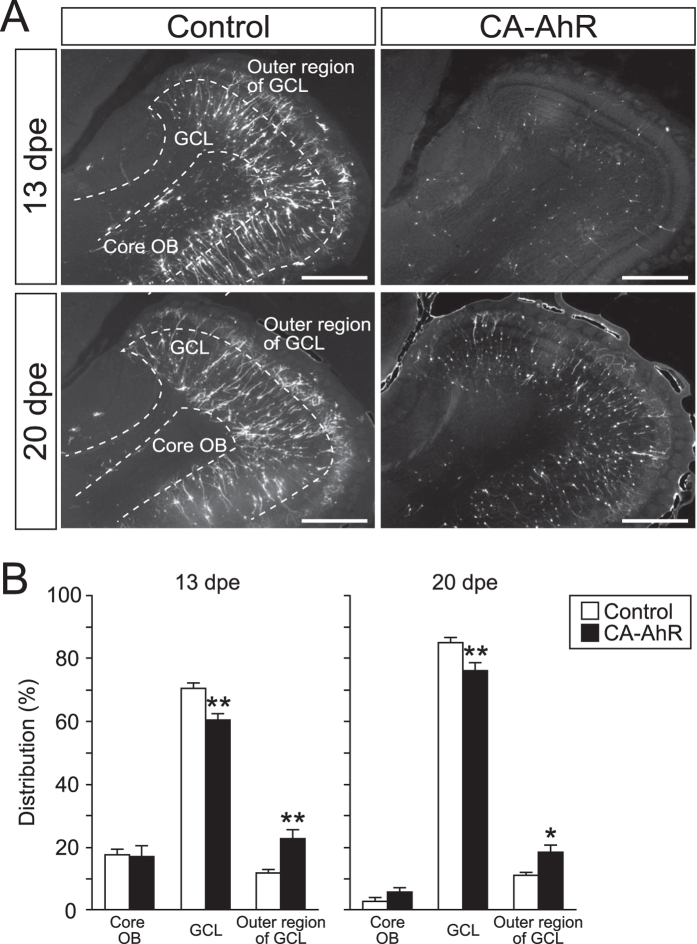
*In vivo* electroporation of CA-AhR led to abnormal distributions of EGFP-expressing cells in the core OB, GCL, and outer region of GCL in the developing mice at 13 and 20 dpe. (**A**) Representative image of EGFP-expressing cells in the GCL of the OB. Scale bar = 500 μm. (**B**) The distribution of EGFP-expressing cells in the GCL and outer region of the GCL, but not the core OB, changed significantly in the CA-AhR group compared with that in the control group at 13 and 20 dpe. Asterisks (* and **): significantly different from the control group at *p* < 0.05 and *p* < 0.01, respectively. Values represent the mean ± standard error of the mean for six mice/group. AhR, aryl hydrocarbon receptor; CA, constitutively active; GCL, granule cell layer; OB, olfactory bulb.

Next, we analyzed the distribution of EGFP-expressing cells to examine radial migration in the OB at 13 and 20 dpe. EGFP-expressing cells were observed in the core OB, GCL, and outer region of the GCL (Fig. 4A). Two-way repeated-measures ANOVA detected significant differences in the interaction between the effect of CA-AhR and the distributions of EGFP-expressing cells in the three regions at 13 and 20 dpe (*p* < 0.001 and  < 0.001, respectively). In the core OB, no statistically significant difference in the percentage of EGFP-expressing cells was observed in the CA-AhR group compared with the control group at 13 dpe (CA-AhR group = 16.9% ± 3.5% vs. control group = 17.5% ± 2.0%, *p* = 0.89) and 20 dpe (CA-AhR group = 5.8% ± 1.5% vs. control group = 3.1% ± 1.1%, *p* = 0.19). In the GCL, the percentage of EGFP-expressing cells in the CA-AhR group was significantly lower compared with that in the control group at 13 dpe (CA-AhR group = 60.5% ± 1.8% vs. control group = 70.7% ± 1.9%, *p* < 0.01) and at 20 dpe (CA-AhR group = 75.9% ± 2.4% vs. control group = 85.7% ± 1.1%, *p* < 0.01). In contrast to the GCL, the percentage of EGFP-expressing cells in the outer region of the GCL was significantly higher in the CA-AhR group compared with that in the control group at 13 dpe (CA-AhR group = 22.6% ± 3.0% vs. control group = 11.8% ± 0.9%, *p* < 0.01) and at 20 dpe (CA-AhR group = 18.3% ± 2.5% vs. control group = 11.2% ± 0.9%, *p* < 0.05; Fig. 4B). These results suggest that the excessive activation of AhR signaling disrupts radial migration in the developing OB.

### Effects of AhR signaling activation on dendritic growth of granule neurons at 13 and 20 dpe

**Figure 5:**
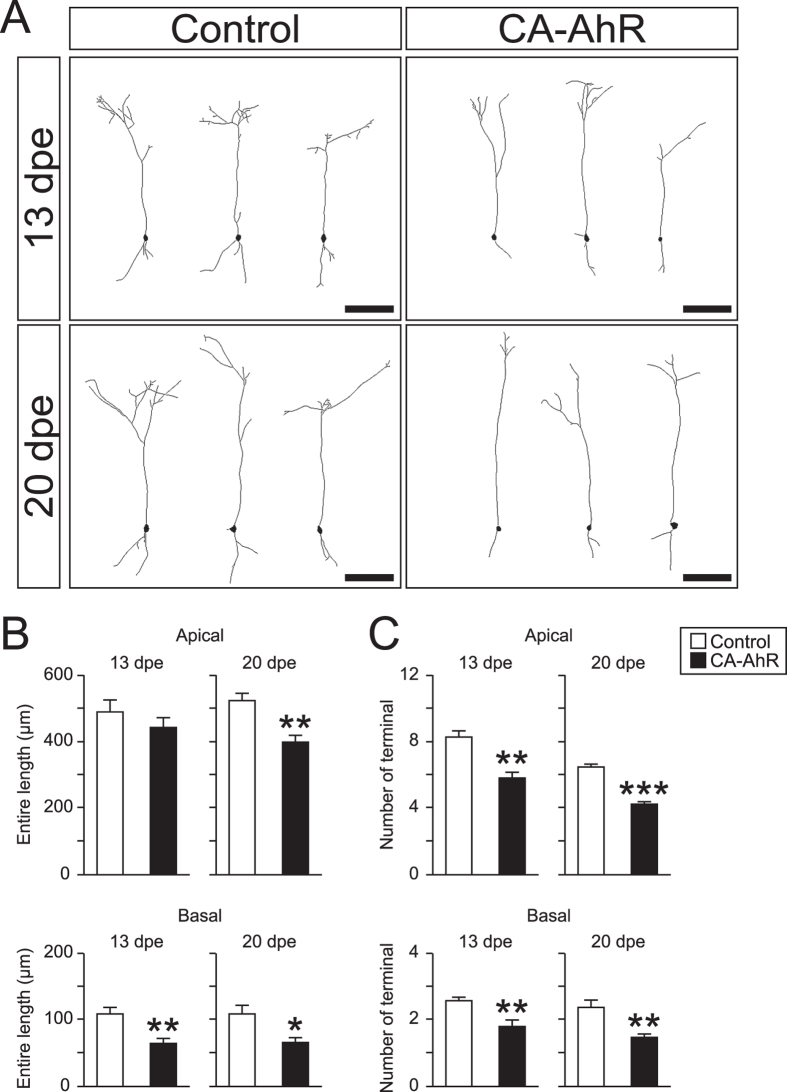
*In vivo* electroporation of CA-AhR reduced the entire length and number of terminals on the apical and basal dendrites of granule neurons in developing mice at 13 and 20 dpe. (**A**) Representative image of EGFP-expressing cells in the granule cell layer of the olfactory bulb at 13 dpe (top) and 20 dpe (bottom). Scale bar = 100 μm. (**B**) Entire length and (**C**) number of terminals on apical and basal dendrites at 13 and 20 dpe. Asterisks (*, **, and ***): significantly different from the control group at *p* < 0.05, *p* < 0.01, and *p* < 0.001, respectively. Values represent the mean ± standard error of the mean for six mice/group.

We analyzed the dendritic morphology of EGFP-expressing cells in the GCL in terms of the entire length of dendrites and the number of terminals (Fig. 5A). The CA-AhR group exhibited a remarkable decrease in the entire length of the basal dendrite (CA-AhR group = 64 ± 8 μm vs. control group = 108 ± 11 μm, *p* < 0.01) but not the apical dendrite (CA-AhR group = 443 ± 32 μm vs. control group = 492 ± 34 μm, *p* = 0.32) at 13 dpe. At 20 dpe, the CA-AhR group exhibited a significant decrease in the entire length of the apical dendrite (CA-AhR group = 397 ± 21 μm vs. control group = 523 ± 21 μm, *p* < 0.01) and the basal dendrite (Fig. 5B; CA-AhR group = 65 ± 8 μm vs. control group = 108 ± 14 μm, *p* < 0.05). The number of terminals on the apical dendrite in the CA-AhR group was significantly lower than that in the control at 13 dpe (CA-AhR group = 5.8 ± 0.4 vs. control group = 8.3 ± 0.4, *p* < 0.01) and at 20 dpe (CA-AhR group = 4.3 ± 0.1 vs. control group = 6.5 ± 0.2, *p* < 0.001). The number of terminals on the basal dendrite in the CA-AhR group was significantly lower than that in the control group at 13 dpe (CA-AhR group = 1.8 ± 0.2 vs. control group = 2.6 ± 0.1, *p* < 0.01) and at 20 dpe (Fig. 5C; CA-AhR group = 1.5 ± 0.1 vs. control group = 2.4 ± 0.2, *p* < 0.01). In addition, the dendritic thickness of granule neurons in the CA-AhR group is much thinner than that in the control group (data not shown). Thus, there is an apparently striking difference in the representative images of the control and CA-AhR groups, as shown in Fig. 4A.

These results suggest that the excessive activation of AhR signaling disrupts the maturation of interneurons in terms of the dendritic growth of apical and basal dendrites.

### CA-AhR induces Cyp1a1 in EGFP-expressing cells

**Figure 6:**
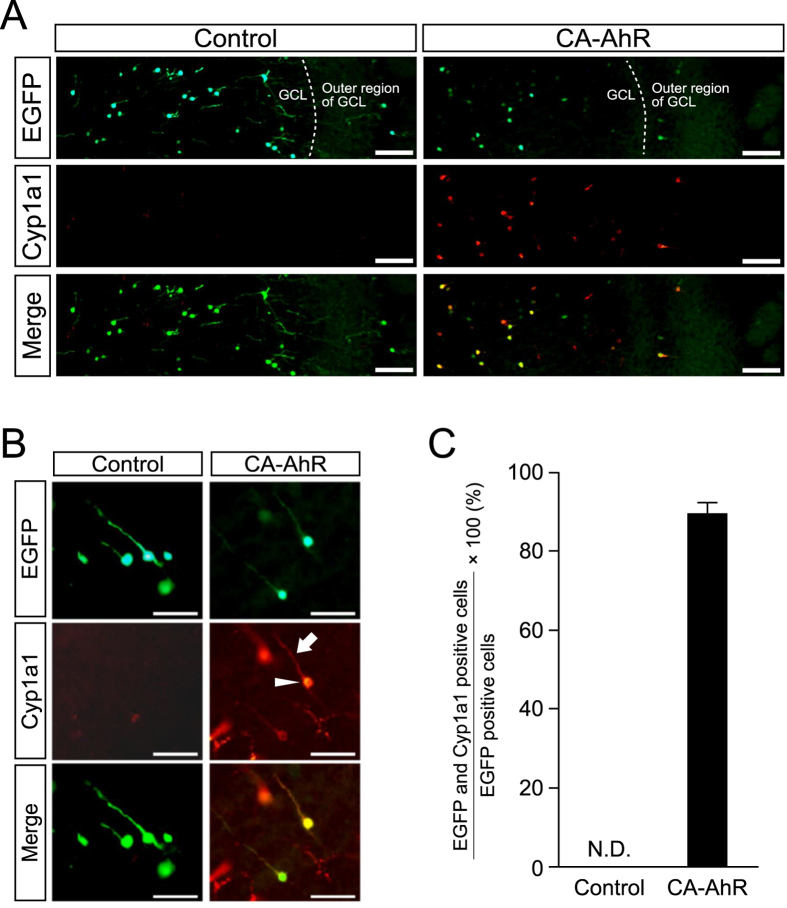
Induction of Cyp1a1 expression in granule neurons in the GCL of the OB at 20 dpe. (**A**,**B**) Representative low (**A**) and high (**B**) magnification photographs showing Cyp1a1 expression in EGFP-expressing cells in the OB in the CA-AhRgroup, but not in the control group. In CA-AhR-electroporated mice, Cyp1a1 signals were observed in the cell body (arrowhead) and apical dendrite (arrow) of granule neurons in the OB. Scale bar = 100 and 50 μm in A and B, respectively. (**C**) Percentages of EGFP and Cyp1a1 double-positive neurons in the total number of EGFP-positive neurons in the control and CA-AhR groups. Values represent the mean ± standard error of the mean for three mice/group. AhR, aryl hydrocarbon receptor; CA, constitutively active; and N.D., not detected.

To examine that the expressed CA-AhR gene can function in mediating AhR signaling, we studied the expression of Cyp1a1, a well-known AhR target marker protein^23^, in brains electroporated with a CA-AhR construct. Distinct Cyp1a1 signals were observed in EGFP-expressing cells in the OB at 20 dpe in the CA-AhR group, but not in the control group (Fig. 6A,B). Quantitative analysis revealed that Cyp1a1 signals were detected in 90% EGFP-expressing cells of the CA-AhR group, but not in the cells of the control group (Fig. 6C). These results suggest that CA-AhR protein can function as a transcription factor to induce AhR signaling in the target cells.

## Discussion

The results of this study provide evidence for the pivotal role of AhR signaling in neuronal differentiation and maturation in the developing brain, where we used olfactory interneurons as a model of GABAergic neurons. An essential role of AhR has been reported for the manifestation of dioxin toxicity and the development of organs in mouse models^13^,^14^,^15^. The physiological roles of AhR in the nervous system have also been demonstrated based on loss-of-function and gain-of-function experiments in invertebrates and vertebrates^2^,^3^,^4^,^5^. In *C. elegans, ahr-1*, a homolog of the mammalian AhR gene, regulates subtype differentiation by GABAergic motor neurons^2^, and the deletion of *ahr-1* induces specific defects in neuronal differentiation, as demonstrated by changes in gene expression, aberrant cellular migration, axonal branching, and supernumerary neuronal processes^5^. *Spineless*, the *Drosophila* homolog of the mammalian AhR, controls the complexity of dendritic arborization in each sensory neuron subtype^3^. In mice, the deletion of AhR reduces cell division, decreases cell survival, and diminishes neuronal differentiation in the dentate gyrus of the hippocampus^4^. Thus, previous studies have demonstrated the crucial role of AhR signaling in the nervous system, but the function of AhR signaling in the developing brain is still unclear for mammalian nervous systems.

In the present study, we showed that the excessive activation of AhR signaling disrupted both tangential migration in the RMS and radial migration in the OB in CA-AhR-electroporated mice (Figs 2 and 4). The tangential and radial migration of neuronal precursors is regulated by β-1 integrin, prokineticin, tenascin-R, and Reelin^29^,^30^,^31^,^32^, thereby suggesting that the expression of these proteins is altered by AhR signaling activation. This hypothesis is supported at least partly by an *in vitro* study, which showed that dioxin exposure induced the expression of β-1 integrin mRNA and protein^36^, although the effects of activated AhR signaling on other molecules are not known. Our morphometric analysis of migrating precursors in the RMS and granule neurons in the GCL detected neurite growth disruption in CA-AhR-electroporated mice (Figs 3 and 5). Doublecortin (DCX) knockdown disrupts the neurite growth of migrating precursors along the RMS enroute to the OB^33^; therefore, our results suggest that DCX expression was disrupted in the CA-AhR-expressing cells. Our experimental findings raise a new question that AhR may take a part in signal cascade that regulates neuronal differentiation and maturation in the developing brain.

Abnormalities in terms of higher brain function, including fear memory, behavioral flexibility, repetitive compulsory responses, and abnormal social behavior, can be induced in mice born to dams exposed to dioxin during gestation^16^,^17^,^18^,^19^,^20^,^21^,^22^. Although the effect of dioxin exposure on the olfactory system is largely unknown, developmental and functional disruption of the OB may involve behavioral abnormalities in dioxin-exposed rodents^16^,^17^,^19^. This notion could be supported by the fact that the olfactory system contains projections into the hippocampus through the entorhinal cortex^37^. In addition, the OB is thought to be associated with higher brain function because olfactory bulbectomy induces behavioral abnormalities, such as the suppression of fear memory^38^. Recently, we reported that the dendritic morphology in the hippocampus was impaired in young mice transfected with CA-AhR, as well as in young and aged mice exposed perinatally to dioxin^23^, and that these morphological disruptions may explain the behavioral abnormalities. In addition, the results of the present study demonstrated the disturbance of cellular migration in interneuron development by AhR signaling activation, which may help to elucidate the developmental neurotoxicity caused by dioxin. Further studies on cellular migration and neurite growth of precursors in the RMS and granule neurons in the OB from dioxin-exposed mice will reveal physiological characteristics, such as AhR expression and AhR signaling activation in these cells. In addition to dioxin, exposure to environmental chemicals, such as methylmercury and lead, during the prenatal period is known to impair brain development and disrupt late-onset higher brain function^39^. Our findings suggest that analyses of the cellular migration and dendritic growth can help confirm behavioral alterations caused by exposure to environmental chemicals.

Dioxin exposure has been reported to induce the signaling pathway for actin polymerization in MCF7 and HepG2 cells^28^,^36^, thereby indicating that cellular toxicity is associated with cytoskeletal regulation. The abnormal neurite morphology induced by CA-AhR (Figs 3 and 5) suggests that AhR signaling activation induces dysregulation of the cytoskeleton, which leads to disruption of cellular migration and dendritic growth, because a number of studies have demonstrated that the cytoskeleton regulates the growth of the leading process and dendrites^35^,^40^. Cyclooxygenase-2 (COX-2), the expression of which can be regulated in an AhR-dependent manner, functions to produce prostanoids (PGs), including PGE_2_^41^. Because PGE_2_ treatment reduces the neurite length of Purkinje neurons and Neuro-2a cells^42^,^43^, it is plausible to speculate that COX-2 expression induced by CA-AhR is involved in the abnormal growth of the leading process and dendrites. Due to the ubiquity of the cytoskeleton in various types of cells, it is possible that CA-AhR may have an impact on the cellular morphology not only in neuronal cells but also in cells in other kinds of organs. Therefore, further studies of the relationship between AhR signaling and cytoskeleton regulation will deepen our understanding of the mechanism of AhR-dependent toxicity.

## Materials and Methods

### Animals

The animal experimental protocol used in this study was approved by the Animal Care and Use Committee of the University of Tokyo, and all experiments were performed in accordance with the relevant guidelines and regulations of the University of Tokyo. Pregnant C57BL/6J mice were purchased from CLEA Japan (Tokyo, Japan). The mice were housed in an animal facility at a temperature of 22–24 °C and humidity of 40–60%, with a 12:12 h light:dark cycle (lights on from 08:00–20:00). Laboratory rodent chow (Lab MR Stock; Nosan, Yokohama, Japan) and distilled water were provided *ad libitum*.

### Vector construction

To produce this CA-AhR cDNA fragment, CA-AhR was amplified by PCR using the specific primers 5′-CTCGAGGCGGGCACCATGAGCAGCGGCGCCA-3′ and 5′-CTCGAGTCAACTCTGCACCTTGCT-3′, with pQCXIN-CA-AhR-EGFP (a kind gift from Dr Yoshiaki Fujii-Kuriyama of Tsukuba University and Dr Tomohiro Ito of the National Institute for Environmental Studies) as a template. The resulting CA-AhR fragment was excised by XhoI digestion and inserted into the XhoI site of pCAGGS1 to generate the pCAGGS1-CA-AhR plasmid. This plasmid DNA was dissolved in 10 mM Tris-HCl (pH 8.0) at a concentration of 2 μg/μl, and Fast Green solution (0.1%) was added to the plasmid solution at a ratio of 1:10, which was used to monitor the injection.

### Neonatal *in vivo* electroporation

Neonatal *in vivo* electroporation was performed as described previously^34^, followed by cellular migration and morphology analyses. On PND 1, male pups were deeply anesthetized by hypothermia (30 s on crushed ice). The plasmid solution was prepared as described and used for neonatal *in vivo* electroporation to induce CA-AhR expression in cells in the RMS, OB, and AOB. For neonatal electroporation, approximately 0.5–1.0 μl of plasmid solution was injected into the lateral telencephalon ventricle of each pup using a hand-made glass micropipette (GD-1; Narishige, Tokyo, Japan), followed by electroporation using a tweezer-type electrode (CUY650-5; Tokiwa Science, Fukuoka, Japan) and an electroporator (CUY21E; Nepa gene, Chiba, Japan). Electric pulses (99 V; 50 ms) were charged four times at 950-ms intervals. Pups electroporated with pCAGGS1-EGFP and pCAGGS1-EGFP + pCAGGS1-CA-AhR were designed as the control and CA-AhR groups, respectively.

### Preparation of brain tissues from electroporated mice

At 6, 13, or 20 dpe, the electroporated pups were anesthetized with sodium pentobarbital and perfused transcardially with 4% paraformaldehyde (PFA) in 0.1 M phosphate-buffered saline (PBS, pH 7.4). The brains were collected and fixed overnight in 4% PFA. Brains were immersed in an ascending series of 5%, 15%, and 30% sucrose in 0.1 M PBS, frozen in Tissue-Tek O.C.T. compound (Sakura Finetek; Tokyo, Japan) and stored at −80 °C until analysis. The RMS and OB tissues were cut with a Leica CM3050S cryostat through the sagittal plane to generate 50-μm thick slices. The tissue sections were collected and rinsed in 0.1 M PBS and placed on slides. Each slide was covered with Vectashield (Vector Laboratories, Burlingame, CA, USA) and a plastic coverslip, before viewing with a Leica DM6000 B microscope.

### Cellular migration analysis

To assess cellular migration in the developing brain, we counted the number of EGFP-expressing cells in the RMS, OB, and AOB regions at 6, 13, and 20 dpe, before comparing the distributions of the EGFP-expressing cells between the regions by calculating the percentage of the cells in each region. At 13 and 20 dpe, we counted the number of EGFP-expressing cells in the core OB, GCL, and outer region of the GCL, and we compared the distributions between the regions in the same manner as described for the RMS, OB, and AOB regions (*n* = 6 mice/group).

### Cellular morphology analysis

The EGFP-expressing cellular structures in the RMS and OB were subjected to morphological analysis using Neurolucida software, which is a computer-based tracing system (MicroBrightField, Colchester, VT, USA). A single cell was traced under a microscope equipped with a specific objective lens (Leica DM6000 B, HCX PL APO, 40×, NA = 0.75; Leica Microsystems). EGFP-expressing cells were observed at 400 times magnification under blinded conditions (i.e., information about the tissue sections was kept from the researcher who analyzed the cellular morphology). The three-dimensional morphology of neurites of EGFP-expressing cells in the RMS and OB was quantified using NeuroExplorer software (MicroBrightField). In the RMS, EGFP-expressing cells were traced singly at 6 dpe. Next, we estimated the angle between the leading processes and the RMS. The number of EGFP-expressing cells analyzed ranged from 25 to 40 neurons per animal. The lengths and number of terminals in the apical and basal dendrites of the granule neurons in the GCL of the OB were quantified at 13 and 20 dpe using NeuroExplorer software.

### Immunofluorescence

To examine the excessive activation of AhR signaling in electroporated cells, we used immunofluorescence to detect Cyp1a1 protein as an AhR-target protein. Thus, frozen tissues were cryosectioned to produce 50-μm thick slices. Anti-Cyp1a1 antibody (sc-9828, Santa Cruz Biotechnology, CA, USA) was used to detect Cyp1a1, as reported previously^23^. Brain sections were washed in 0.01% Triton X-100 in PBS (PBST) and soaked in 1 mM EDTA solution (pH 8.0), before treating in a microwave at 600 W for 90 s. After incubation with 10% bovine serum for blocking, the tissue sections were incubated with the first antibody (anti-Cyp1a1, 1:100). Finally, the signals were visualized with the second antibody (anti-Goat IgG AlexaFluor 568 conjugate, Life Technologies, MD, USA), before washing in PBST and mounting for fluorescence microscopy.

### Statistical analysis

All values were expressed as the mean ± standard error of the mean, and the Student’s *t*-test was used for statistical analysis. A two-way ANOVA was performed to compare the cellular distributions and leading process directions between the control and CA-AhR groups. *p* < 0.05 was considered statistically significant.

## Additional Information

**How to cite this article**: Kimura, E. *et al*. AhR signaling activation disrupts migration and dendritic growth of olfactory interneurons in the developing mouse. *Sci. Rep.* **6**, 26386; doi: 10.1038/srep26386 (2016).
